# Supplementary material for: Healthcare Resource Utilization, Economic Burden, and Multi-Level Medical Security System for Individuals with Spinal Muscular Atrophy in Shaanxi Province, China
Source: Healthcare (Basel). 2025 Feb 17;13(4):428. doi: 10.3390/healthcare13040428 (PMC11855159; doi:10.3390/healthcare13040428)
Supplement: Supplementary file 1 [file healthcare-13-00428-s001.zip › healthcare-3433285-supplementary.pdf]

Supplementary Table S1: A multi-tiered medical insurance system in China

First tiered	Second tiered	Third tiered
✓Basic Medical Insurance	✓Supplementary Medical Insurance	✓Social Assistance System
· Basic Medical Insurance for Urban Employees	· Enterprise Supplementary Medical Insurance	· Charitable Aid
· Basic Medical Insurance for Urban and Rural Residents	· Commercial Health Insurance	· Social Assistance
✓Critical Illness Insurance		· Social Welfare
✓Medical Assistance		· Preferential Treatment and Resettlement
✓Long-term Care Insurance		

Supplementary Table S2: The mean cost(In CNY and 1 CNY = 0.14 USD) of Basic Medical Insurance Reimbursement for nusinersen injection per SMA patient in Shaanxi province, China

Year	No. of patient	Total cost	Basic Medical Insurance	Critical Illness Insurance	Medical Assistance	Out-of-Pocket
2021	2	833034.47	764.37	0	0	832270.10
2022	34	188332.95	98910.04	35748.15	1310.66	52364.11
2023	26	160839.09	87681.75	30058.85	2970.41	40128.08
